# The Convention on the rights of persons with disabilities and newborns/children

**DOI:** 10.1186/1824-7288-41-S2-A40

**Published:** 2015-09-30

**Authors:** Giampiero Griffo

**Affiliations:** 1 World Council of Disabled Peoples’ International (DPI), Napoli, Italy

## 

The Convention on the rights of persons with disabilities of United Nations (CRPD 2006) is an international standard, but not enough know in Italy. The CRPD introduce a strong change of paradigma in the approach on persons with disabilities. Based on the respect of human rights, is innovative regard the Convention on the rights of the child (1989). Redefining the concept of disability, stress the importance of the social and relationship components. For the minors with disabilities means a new responsibility of the pediatrics on the social components that can determine factors of impoverishment or empowerment of capacities and competences. New concepts (related to new treatment) are stressed: from the empowerment to habilitation and to capability. The relationship with the family too from the curative attention, often totally transfer to specialism, to educational responsibility, guiding to an aware approach of the role of caregiver support, but no substitutive of the possible self-determination. The role of the physicians changes: no more search the recovery, often impossible, but the appropriate support to the empower and habilitate the form of fonctioning of the minor. The “noting about us without us” is essential for the evolution of minors with disabilities, put in value the human diversities, that are part of the human kind.

